# Global Infectious Disease Surveillance and Case Tracking System for COVID-19: Development Study

**DOI:** 10.2196/20567

**Published:** 2020-12-22

**Authors:** Hsiu-An Lee, Hsin-Hua Kung, Yuarn-Jang Lee, Jane C-J Chao, Jai Ganesh Udayasankaran, Hueng-Chuen Fan, Kwok-Keung Ng, Yu-Kang Chang, Boonchai Kijsanayotin, Alvin B Marcelo, Chien-Yeh Hsu

**Affiliations:** 1 Department of Computer Science and Information Engineering Tamkang University New Taipei Taiwan; 2 Taiwan e-Health Association Taipei Taiwan; 3 Asia eHealth Information Network Hong Kong Hong Kong; 4 Standards and Interoperability Lab Smart Healthcare Center of Excellence Taipei Taiwan; 5 Department of Information Management National Taipei University of Nursing and Health Sciences Taipei Taiwan; 6 Division of Infection Diseases Department of Internal Medicine Taipei Medical University Hospital Taipei Taiwan; 7 Nutrition Research Center Taipei Medical University Hospital Taipei Taiwan; 8 Master Program in Global Health and Development College of Public Health Taipei Medical University Taipei Taiwan; 9 Sri Sathya Sai Central Trust Prasanthi Nilayam Puttaparthi India; 10 Department of Rehabilitation Jen-Teh Junior College of Medicine, Nursing and Management Miaoli Taiwan; 11 Department of Medical Research Tung’s Taichung Metroharbor Hospital Taichung Taiwan; 12 Department of Pediatrics Tung’s Taichung Metroharbor Hospital Taichung Taiwan; 13 eHealth Research Institute Hong Kong Hong Kong; 14 Thai Health Information Standards Development Center Health System Research Institute Ministry of Public Health Bangkok Thailand; 15 University of the Philippines Manila Philippines

**Keywords:** blockchain, infectious disease surveillance, international collaboration, HL7 FHIR, COVID-19 defense, COVID-19

## Abstract

**Background:**

COVID-19 has affected more than 180 countries and is the first known pandemic to be caused by a new virus. COVID-19’s emergence and rapid spread is a global public health and economic crisis. However, investigations into the disease, patient-tracking mechanisms, and case report transmissions are both labor-intensive and slow.

**Objective:**

The pandemic has overwhelmed health care systems, forcing hospitals and medical facilities to find effective ways to share data. This study aims to design a global infectious disease surveillance and case tracking system that can facilitate the detection and control of COVID-19.

**Methods:**

The International Patient Summary (IPS; an electronic health record that contains essential health care information about a patient) was used. The IPS was designed to support the used case scenario for unplanned cross-border care. The design, scope, utility, and potential for reuse of the IPS for unplanned cross-border care make it suitable for situations like COVID-19. The Fast Healthcare Interoperability Resources confirmed that IPS data, which includes symptoms, therapies, medications, and laboratory data, can be efficiently transferred and exchanged on the system for easy access by physicians. To protect privacy, patient data are deidentified. All systems are protected by blockchain architecture, including data encryption, validation, and exchange of records.

**Results:**

To achieve worldwide COVID-19 surveillance, a global infectious disease information exchange must be enacted. The COVID-19 surveillance system was designed based on blockchain architecture. The IPS was used to exchange case study information among physicians. After being verified, physicians can upload IPS files and receive IPS data from other global cases. The system includes a daily IPS uploading and enhancement plan, which covers real-time uploading through the interoperation of the clinic system, with the module based on the Open Application Programming Interface architecture. Through the treatment of different cases, drug treatments, and the exchange of treatment results, the disease spread can be controlled, and treatment methods can be funded. In the Infectious Disease Case Tracking module, we can track the moving paths of infectious disease cases. The location information recorded in the blockchain is used to check the locations of different cases. The Case Tracking module was established for the Centers for Disease Control and Prevention to track cases and prevent disease spread.

**Conclusions:**

We created the IPS of infectious diseases for physicians treating patients with COVID-19. Our system can help health authorities respond quickly to the transmission and spread of unknown diseases, and provides a system for information retrieval on disease transmission. In addition, this system can help researchers form trials and analyze data from different countries. A common forum to facilitate the mutual sharing of experiences, best practices, therapies, useful medications, and clinical intervention outcomes from research in various countries could help control an unknown virus. This system could be an effective tool for global collaboration in evidence-based efforts to fight COVID-19.

## Introduction

COVID-19, which presumably originated in bats and was transmitted to humans by means of unknown mechanisms in Wuhan, Hubei Province, China in December 2019, has affected more than 180 countries and territories around the world. On March 11, 2020, the World Health Organization (WHO) characterized the COVID-19 outbreak as a pandemic. This is the first pandemic known to be caused by a new virus. Although the complete clinical picture with regard to COVID-19 is not fully known, based on currently available information, older adults and people with serious underlying medical conditions might be at a higher risk for the severe illness caused by COVID-19.

Since a total of 41 cases with an unknown etiology of pneumonia were confirmed in Wuhan City, Hubei Province, China in December 2019 [1], COVID-19 has spread rapidly across that country and around the world [2-8]. Thus far, it has affected more than 12,723,798 people in 188 countries and regions (data obtained through July 12, 2020) [9]. COVID-19 is now the most serious infectious disease event after severe acute respiratory syndrome (SARS) in 2003, and no effective vaccine, drug, or treatment has been found.

Many different infectious diseases still exist in the world, such as the Ebola hemorrhagic fever, the highly pathogenic avian influenza, SARS, Middle East respiratory syndrome (MERS)–related coronavirus, and seasonal influenza. When an infectious disease event occurs suddenly, it is crucial to find a quick treatment and control method. Normal patient treatment needs to be based on the medical history and symptoms of the different cases.

The rise of COVID-19 was sudden and marked by the global information flow not being fast enough and the case reports being transmitted slowly, which has led to a sluggish treatment progress, patients not being cured in an efficient manner, and the infectious disease still not being effectively controlled. In today’s age of information, our global connectivity gives us a strong advantage in the fight against infectious diseases. We can analyze large amounts of data to identify outbreaks across different parts of the world, and we can use advanced machine learning models to predict their future movement across different geographical territories. The challenge is that collating relevant data and standardizing it on a global level is a complicated task. In many parts of the world, data does not flow easily from hospitals into the public realm or across borders. Global data standards have yet to be developed, and this creates gaps in the data sets and delays in how the data can be used to shape global health efforts. One way of improving the speed that data is standardized could be to encourage better interconnectivity across national data systems by using more homogenous data standards. This would require a great deal of collaboration between the various stakeholders, and it could be challenging to promote it across borders [10].

The challenge of a slow and insufficient global information flow could be tackled by a good framework such as the Asia eHealth Information Network’s Governance, Architecture, Program Management, Standards and Interoperability framework as well as a good collaboration model.

According to different research case reports in China [2,5,6], of the patients who are in the 18 years and older group, 61.9% (n=172) were male, and in another report, 2 of 13 patients with COVID-19 were children, who ranged between 2 and 15 years old [11]. Conclusions of the symptoms and disease history of patients with COVID-19 were found in these studies. Hypertension and cardiovascular disease were the two most common diseases in the adult patient group, followed by diabetes mellitus. With regard to the symptoms, fever was the most common (n=28, 92.8%), followed by a cough (n=194, 69.8%), dyspnea (n=96, 34.5%), myalgia (n=77, 27.7%), a headache (n=20, 7.2%), diarrhea (n=17, 6.1%), a sore throat (5.1% [6]), and pharyngeal (17.4% [2]). Wang et al [2] showed that the intensive care rate was significant in older patients. Other research noted that patients who needed intensive care had a greater percentage of dyspnea than those not needing intensive care [2,5]. From a report presented by a Beijing research team, among 13 patients with COVID-19, 12 (92.3%) had a fever, with a mean of 1.6 days before the patient went to a hospital, and they had a cough (46.3%), myalgia (23.1%), upper airway congestion (61.5%), and a headache (23.1%) [11].

Although there are many reports and studies on COVID-19, the details of disease control and treatment are still being broadcast slowly, which may cause the disease spread to be out of control and make it difficult to share the experiences of successful case treatments. According to the control status and experience of COVID-19, all cases should be uploaded to the WHO website by different governments, but the route of transmission is still difficult to track, and treatment experiences in different countries cannot be effectively shared. A literature review of infectious disease surveillance, presented by Jajosky and Groseclose [12], and an analysis of the timeliness of reporting by the National Notifiable Diseases Surveillance System showed that longer reporting lags and the variability among the states limit its usefulness. Some systems have the function of being a static continuous spatial map of infectious disease risk, while others have the function of continuously updating the reporting of infectious diseases, but there is still no system that combines these two functions [13].

After the rise of COVID-19, the problem has developed into the pathogenic spread across, and among, nations by means of international travel, which has unfortunately enabled the pathogens to invade new countries and adapt to new environments and hosts faster [14,15]. In many countries where the public health infrastructure is poor or where there is an insufficient budget to develop it, the ability of electronic disease surveillance, including data collection and an analysis capability, should be improved [16,17]. Furthermore, the data exchange of international infectious disease reports and information has certain constraints, not only out of fear for the repercussions on trade and tourism but also because of the delays in data transfer through the multiple levels of governments or organizations [18]. After experiencing epidemic infectious diseases caused by mutant viruses such as SARS and MERS, we have found that, when facing treatment for unknown diseases, related health organizations and authorities should conduct comprehensive tests, using different drugs and treatment methods, and they should then present the differences between each case and the analyzed treatment results to find the best treatment. However, this process is tedious and dangerous, and it creates uncertainties regarding patient treatment. In the face of new infectious diseases, the exchange of treatment results and case experiences is critical.

When facing a new type of infectious disease, it is important not only to treat the disease but also to prevent its contagion. For example, hundreds of COVID-19 cases in South Korea were found to have occurred at the same church. Hundreds of cases in Japan were found to have originated on a cruise ship. In Hong Kong, several cases were found to have been infected through a hot pot meal. Iran’s speedy and large-scale infection may be due to specific types of religious behavior. In Italy, the outbreak may have been caused by the Italian culture, where hugs and kisses are a common way of greeting someone. During the SARS outbreak in 2003, it was found that infections were caused by the drainage designs of high-rise buildings [19]. Information on the correlation between the context of the event, living, transportation or environmental design, religion, and cultural behavior is critical for studying COVID-19 transmission.

To understand the epidemiology and trends of COVID-19, the WHO has provided a template for a case-based reporting form and a data dictionary for that case-based reporting form, and it has requested member countries to report probable and confirmed cases of COVID-19 infection within 48 hours of their identification [20]. These reports are sent through the National Focal Point and the Regional Contact Point for International Health Regulations at the appropriate WHO regional office. The WHO has asked the countries to provide aggregated data for surveillance when it is not feasible to report case-based data.

However, to the best of our knowledge, there has thus far been no functional collaborative global case exchange model that can cocreate case data on COVID-19 and facilitate care coordination across countries. The aim of this study is to design an infectious disease surveillance module for the global exchange of infectious cases and the sharing of treatment experience. Information on the movement and path tracking of cases, including the linkage and correlation between each case, can also be included in infectious disease control in various countries. Therefore, when an infectious disease outbreak occurs, it can be quickly controlled.

In the initial stages of the COVID-19 outbreak, little research was available on the data format of the disease, and no one knew what the best data format was; there had only been some discussions on the importance of clinical data exchange regarding the disease.

Currently, several places have created a Fast Healthcare Interoperability Resources (FHIR)–based COVID-19 data structure. A good example is provided by the National Coordinator for Health Information Technology in its Interoperability Standards Advisory section of Interoperability for the COVID-19 Novel Coronavirus Pandemic [21,22], namely, the Logica COVID-19 (FHIR v4.0.1) Implementation Guide CI Build. The Logica used Health Level 7 (HL7) FHIR profiles for COVID-19 to create an implementation guide for a collection or library of data elements that relate to COVID-19. This can be used in many different situations where COVID-19 data are shared to support patient care, billing, research, or public reporting.

Another example can be found in the Dedalus COVID-19 Solution [23]. In their “COVID-19 Simplifier Project,” they used FHIR resources in the Dedalus COVID-19 Solution software. The data elements cover a patient self-assessment, a remote clinical assessment, and telemedicine and self-monitoring. They claimed that their first activations will be in Italy and France. Our study uses a similar method that started from COVID-19–related clinical data, and we used the International Patient Summary (IPS) as a basis for the data structure. The IPS document is an electronic health record (EHR) extract that contains essential health care information for the necessary care of patients. Due to the rapid outbreak of the disease in the early weeks, no format had been designed for the exchange of COVID-19 data. Therefore, we designed a version of the IPS that can be used for COVID-19.

An IPS document is an EHR extract that contains essential health care information about a patient [24]. It is designed to support the used case scenario for *unplanned, cross-border care*, but it is not limited to that. It is intended to be international (ie, to provide generic solutions for global application beyond a particular region or country), and the IPS data set is minimal and nonexhaustive, specialty agnostic, and condition independent yet still clinically relevant. The design, global scope, and utility of IPS toward unplanned cross-border care, and its potential for reuse, make it suitable for a situation like COVID-19. The FHIR confirmed that IPS, including the symptoms, therapies, medications, and laboratory data, can be efficiently transferred and exchanged on the system for easy access by physicians. Patient data are deidentified to protect their privacy. In addition, the blockchain-based architecture can be used to ensure the security and immutability of the case data.

Our goal is to provide an immediate reference for people to use in the current crisis, so the design is not focused on a single use case, and the IPS therefore has a more general data structure that focuses on the clinical data needed for COVID-19.

We understand that the data structure will not be perfect or comprehensive, but it can be modified in the future after more and more institutions use the data structure to exchange records. According to the research of Holmgren et al [25], the inability of hospitals to receive electronic data is an obstacle for the effective monitoring of patient symptoms. Therefore, the aim of our study is to create a COVID-19 data structure and a system that can share the data among health care institutions. It is expected that the proposed system can contribute to the control of the COVID-19 situation.

## Methods

### Architecture for the Global Infectious Disease Surveillance and Case Tracking Model

**Figure 1:**
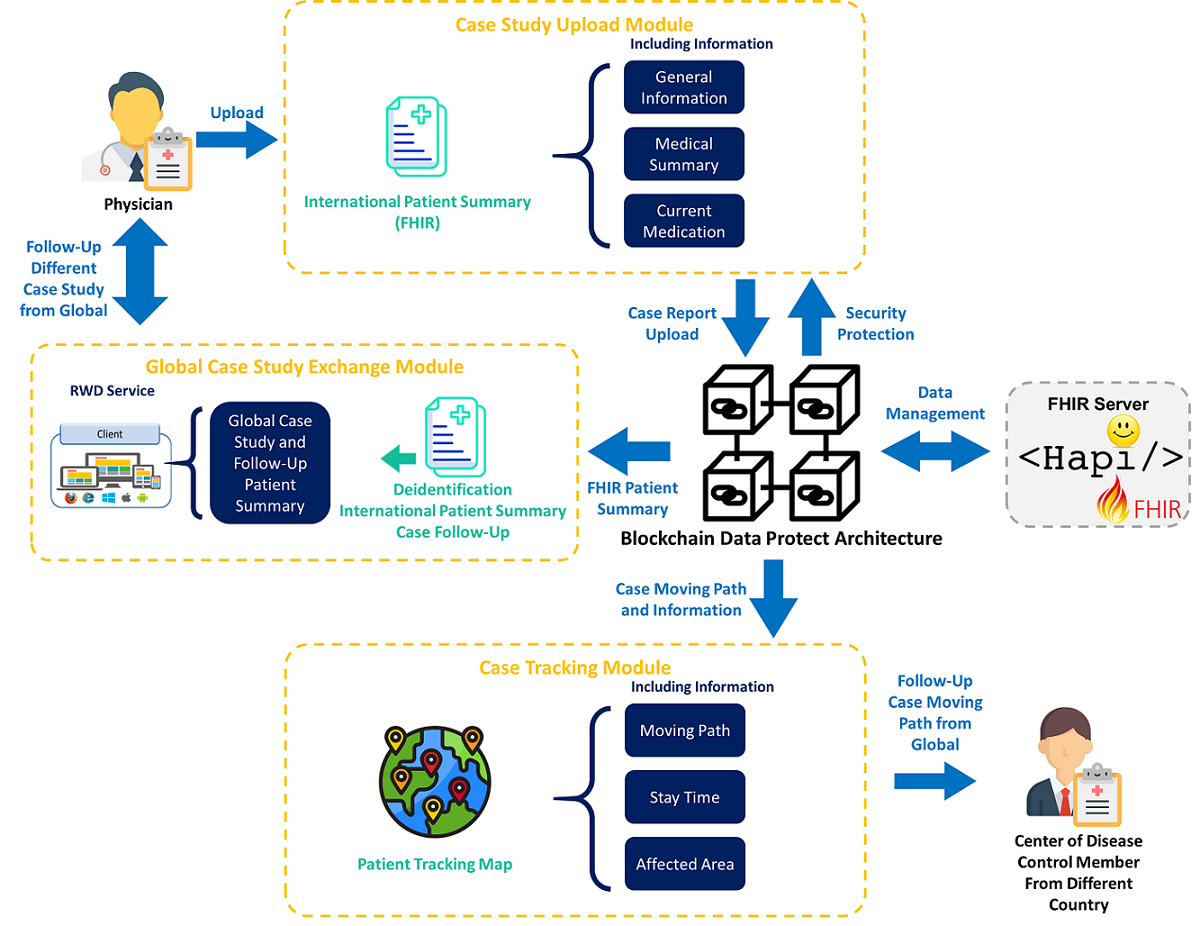
Architecture for the Global Infectious Disease Surveillance and Case Tracking module. FHIR: Fast Healthcare Interoperability Resources; RWD: Responsive Web Design.

This study designs a global infectious disease surveillance and case-tracking model, and it includes a “Case Study Upload Module,” a “Global Case Study Exchange Module,” and a “Case Tracking Module.” Each module has different goals. The architecture of the global infectious disease surveillance and case-tracking model is shown in Figure 1.

The main goal of the “Case Study Upload Module” is to allow physicians worldwide to continuously upload the IPS documents and to include detailed information about the treatment of patients with COVID-19. Through sharing experience and patient summaries with other physicians, they can find better essential treatment methods. This module has the ability to identify and verify the identity of physicians in different countries or regions. The “Global Case Study Exchange Module” allows physicians to brainstorm together on different patient summaries and to learn about, and find, possible potential treatments. A large amount of open and complete information is required for currently unsolved disease treatment issues. Under the condition of privacy protection and the provision of correct information with regard to the different case symptoms, treatment methods, drugs, etc, it may be possible to find the best antidote to solve the infectious disease crisis the world is facing. The “Case Tracking Module” allows Centers for Disease Control and Prevention (CDC) members to track a patient’s movement path before a diagnosis is made. The tracking map is shown in the module. According to different patients’ statements about their own moving paths, a moving map can be established that contains international paths. CDC members will be able to carry out risk control and track high-risk groups according to this map, thereby effectively controlling the scope of disease infection and completing it as soon as possible.

The security and correctness of the IPS are protected by blockchain architecture. When IPS data are uploaded, the details of the data will be deidentified, the block will store the data update log, and the IPS hash value is calculated by the Secure Hash Algorithm (SHA)-256. The IPS data are stored in the HAPI FHIR database, which is open source and an implementation of the interoperability of HL7 FHIR for health care systems in Java. It was developed as an open community by a global team [26]. The IPS continuity of each patient will be connected through the information of the blockchain. User identities are divided into two types, namely, physicians and CDC members. Physicians need to be authenticated through their medical ID certificate in their countries, and CDC members are registered and managed by the CDC units in various countries.

### The IPS Tailored for COVID-19 Case Data

An “International Patient Summary Implementation Guide” has been published by HL7 FHIR. The goal is to provide a universal international solution for global health care service applications. This study uses the IPS (Standard for Trial Use 1-FHIR R4, launched on August 6, 2019) as a case study, as it provides treatment and health care information records for global cases of unknown infectious diseases. IPS is a minimal and nonexhaustive patient summary, which means that it is not intended to copy the full content of an EHR. The IPS is usable by clinicians for the unscheduled cross-border care of a patient and focuses on a patient’s current condition, instead of anything specific to a particular condition. Furthermore, the IPS is applied on a global scale to address the international feasibility of use as much as possible.

**Figure 2:**
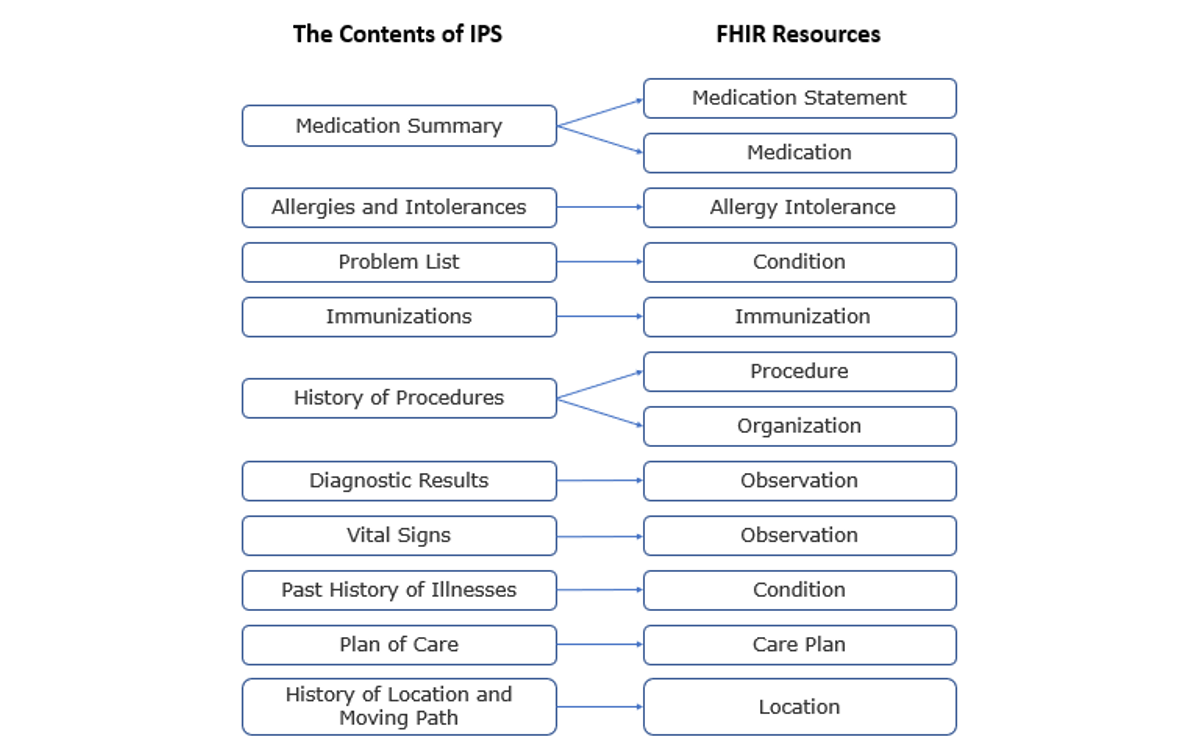
IPS contents mapped to the structures of FHIR resources. FHIR: Fast Healthcare Interoperability Resources; IPS: International Patient Summary.

To provide a reference for global cases, the IPS is designed to include information on the following: “Medication Summary,” “Allergies and Intolerances,” “Problem List,” “Immunizations,” “History of Procedures,” “Diagnostic Results,” “Vital Signs,” “Past History of Illness,” “Plan of Care,” “History of Location and Moving Path before Diagnosis,” and “Location.” The structure of the IPS is shown in Figure 2.

### Case Study Upload Module for IPS Protection and Validation

**Figure 3:**
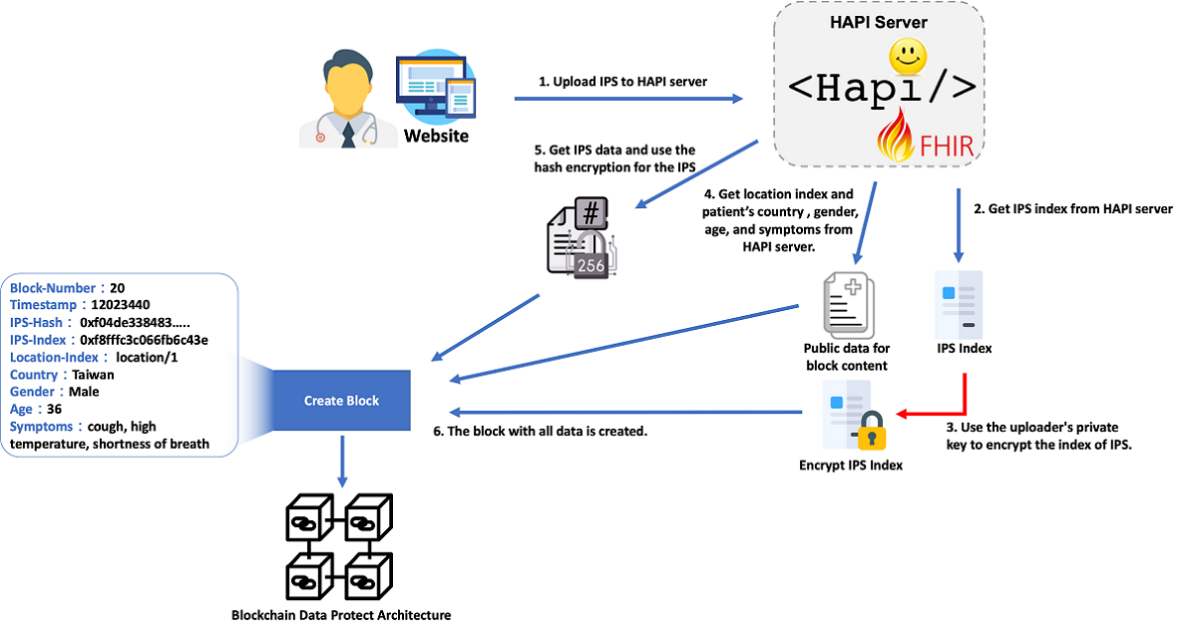
The encryption and decryption for the data upload process and architecture. FHIR: Fast Healthcare Interoperability Resources; IPS: International Patient Summary.

The physician uploads the patient’s IPS document to the system’s HAPI server, and the HAPI server corresponds to the IPS index with the blockchain architecture. The IPS index information was designed to connect the data from the HAPI server, including the IPS hash value and the encrypted IPS index value. The deidentified and simplified case data include the gender, age, symptoms, country, and location index value of the HAPI server. After the physician has been authenticated, they have permission to upload the IPS document and view its study cases. The encryption and decryption for the data upload process and architecture is shown in Figure 3.

The steps of this process are as follows:

- Step 1: The certified physician uploads the patient’s IPS file to the system, and the IPS file will be stored in the HAPI server. Patient identification will be replaced by a globally unique identifier (GUID), which is an 128-bit number that is used to identify the information in the system.
- Step 2: The data index position of the IPS is obtained from the HAPI server.
- Step 3: The private key of the uploaded physician is used to encrypt the IPS index of the data, which is stored in the HAPI server.
- Step 4: The anonymous IPS public information is obtained from the HAPI server, including the mobile path index position, gender, age, country, and symptoms.
- Step 5: The hash value of the IPS file is calculated by the SHA-256 encryption function.
- Step 6: The content of this block is transferred to the blockchain architecture, and a new block is established by the blockchain architecture.

### Global Case Study Exchange Module

In a state of globalization, new diseases or clinical pathways that are not treated correctly are likely to rage around the world. COVID-19 has spread worldwide, and therapeutic vaccines and drugs have not yet been developed to treat it. This study constructed a global patient summary exchange model and shared the global research progress through case analyses so that physicians in different regions of the world can refer to the results of acquisition and test cases while at the same time obtaining and learning more about the unknown disease and finding the best treatment process.

**Figure 4:**
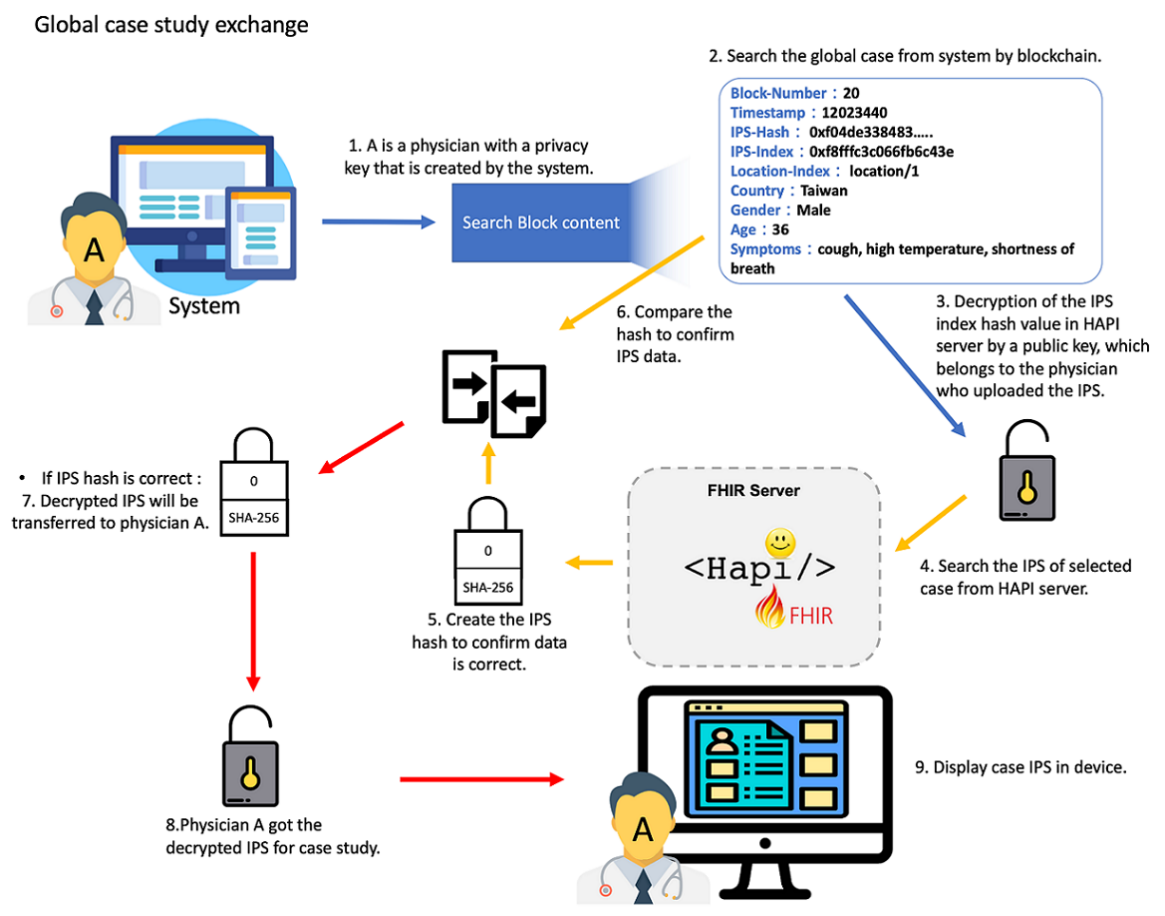
Process of physicians getting the IPS files of global case studies. FHIR: Fast Healthcare Interoperability Resources; IPS: International Patient Summary; SHA: Secure Hash Algorithm.

Our study is designed for IPS sharing, which can help clinical physicians to find successful treatments and clinical pathways to improve the patients’ survival and reduce sequelae. We have designed the model so that physicians need to register first and provide proof of their identity. The system provides each physician with a privacy key for IPS decryption. This system allows physicians to view the summary of the patient cases that have been uploaded all over the world, and it provides a filter function of the cases. Specific cases can be tracked by using this module. The process of how physicians get the IPS files of global study cases is shown in Figure 4.

We designed a nine-step process for completing the Systems Engineering Initiative for Patient Safety (SEIPS) access to international cases, which includes a data search, decryption, verification, and transmission.

- Step 1: The system verifies the identity of the user, confirming that the user is a physician with registration data.
- Step 2: A list of global patients and simple case information is provided to the physicians, including the patient’s region, country, age, and gender.
- Step 3: The index of the selected SEIPS is decrypted by the privacy key of the physician who is uploading the IPS file.
- Step 4: The selected patient IPS file is retrieved from the decentralized database.
- Step 5: The decrypted IPS data are hashed again by SHA-256.
- Step 6: The hash value that is decrypted in step 5 is compared to the hash value in the blockchain.
- Step 7: If the two hash values are equal, it means the data are correct, and the decrypted data are transmitted to the physician.
- Step 8: The system confirms that the physician has obtained the decrypted case study data.
- Step 9: All the IPS files of the selected cases are presented on the physician’s display.

The module is designed as a web-based application, and it includes the Open Application Programming Interface (API) architecture. The module provides various APIs to let the public and private physicians’ clinic management system operate easily with the module and to conduct the case exchange.

### Case Tracking Module for Infectious Disease Prevention

The prevalence of international tourism and the rapid movement of populations, in an era of globalization, have increased the spread of COVID-19. In just 3 months, it has spread from a limited area (one city in Asia) to becoming a source of infection throughout the world, and the number of infected people continues to increase.

**Figure 5:**
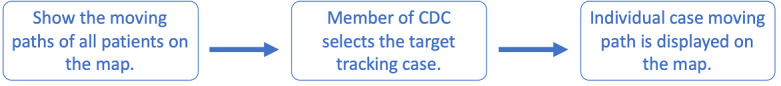
Workflow of case tracking. CDC: Centers for Disease Control and Prevention.

To effectively control the scope of infection and prevent continued expansion, the movement path of patients who are infected needs to be tracked. The FHIR “Location” resource is included in the patient’s IPS file, and it helps CDC members effectively track the patients and prevent the continued spread of the disease, based on the record of moving paths and time stamps. The workflow of case tracking is shown in Figure 5.

### Structure of Blockchain Security

The blockchain architecture was established as the security protection mechanism of IPS data, and the HAPI server was used as a data server for the FHIR IPS. The block in the blockchain is public data for all users and includes the IPS index information and deidentified simple case data, which includes gender, age, symptoms, country, and the HAPI server data index.

Blockchains have many different authority mechanisms. In this study, considering the privacy of a patient’s medical data and the need to process a large amount of medical information, the blockchain was built in a private chain, and a Proof of Authority (PoA), with a fast transaction speed and high privacy, was adopted as the consensus on the blockchain. In 2015, PoA was proposed by the Ethereum cofounder, Gavin Wood [27]. This consensus algorithm is used to set up trusted nodes as block validators. It is a centralized consensus mechanism that ensures data security and data verification through authorization mechanisms. The blocks on the chain are generated by trusted nodes, which can improve the efficiency of the generating blocks and ensure consistent data. At the mean times, the system runs well. The ownership of the nodes depends on the policy of the health care authority in different areas. For example, it can be a hospital center or the CDC of a nation.

**Figure 6:**
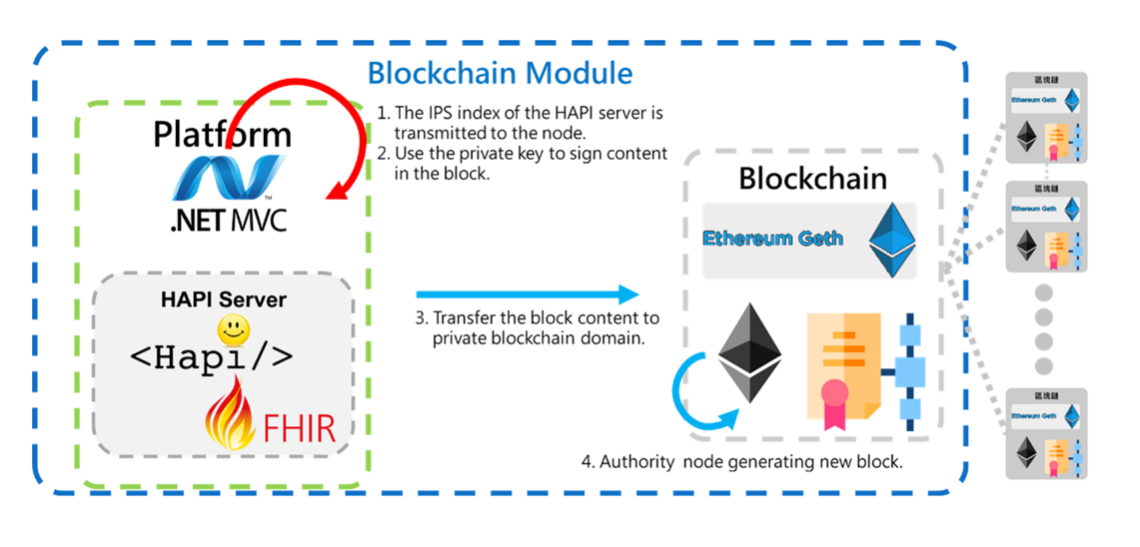
The process of generating a new block. FHIR: Fast Healthcare Interoperability Resources; IPS: International Patient Summary; MVC: model–view–controller.

The process of generating a new block includes four steps, as shown in Figure 6.

- Step 1: The IPS index of the HAPI server is transmitted to the node.
- Step 2: The private key is used to sign the content in the block.
- Step 3: The block content is transferred to the private blockchain domain.
- Step 4: The authority node generates a new block.

Blockchain architecture will automatically copy the new block data to other nodes to complete the goal of blockchain decentralization.

## Results

### Global Infectious Disease Surveillance of the IPS for Case Studies

When facing the spread of an unknown disease around the world, such as COVID-19, global case studies must be shared and exchanged quickly. Clinical data must be allowed to be transmitted efficiently and safely to jointly find the most appropriate control and treatment methods through international cooperation. Because different patients have different disease histories, family disease histories, and life environments, their symptoms and disease progression will be different.

An example of this is the SARS outbreak in 2003. After the outbreak, Hong Kong found numerous problems in the surveillance systems of communicable diseases, and the 2003 contact tracking system was inadequate for dealing with the scale of the SARS epidemic. The public health surveillance systems were not well developed in the private sector and in community clinics, there was no comprehensive laboratory surveillance system, and the hospital authority’s laboratory database was not linked to the department of health in the early stages of the epidemic.

**Figure 7:**
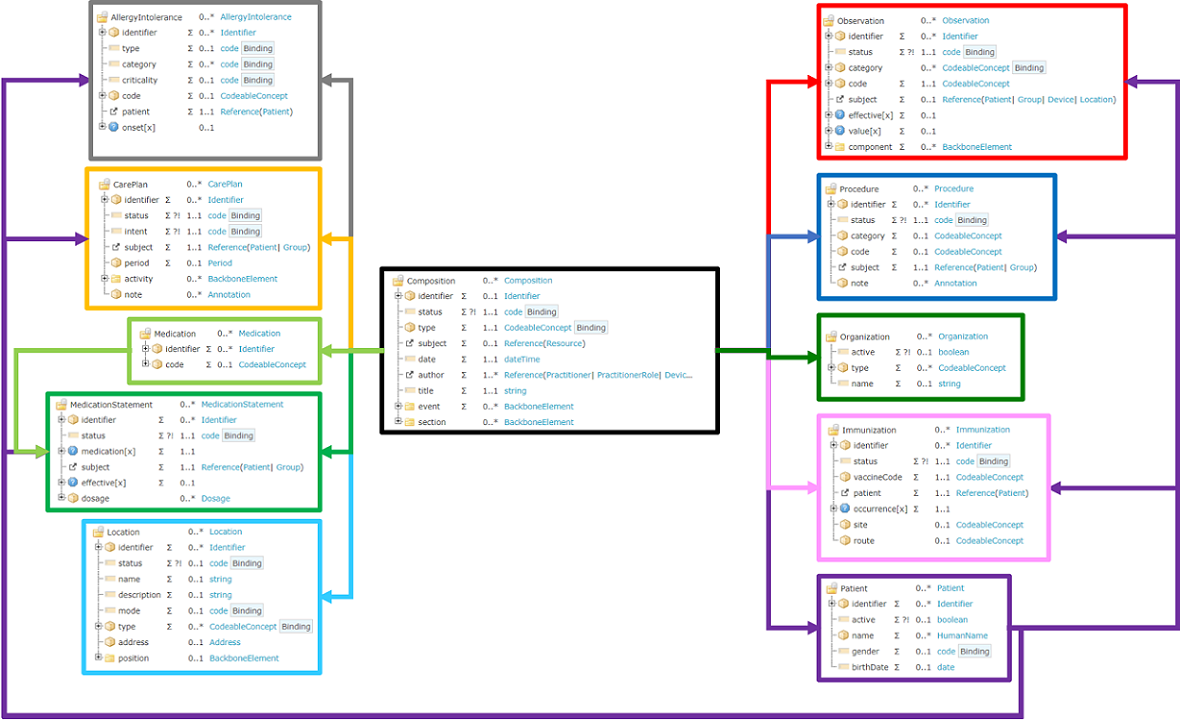
International Patient Summary contents are mapped to the structures of the Fast Healthcare Interoperability Resources.

This study thus designs an IPS that complies with infectious disease surveillance and clinically meaningful data, according to the IPS HL7 FHIR guidelines. The IPS that we designed includes the following: “Medication Summary,” “Allergies and Intolerances,” “Problem List,” “Immunizations,” “History of Procedures,” “Diagnostic Results,” “Vital Signs,” “Past History of Illness,” “Plan of Care,” and “History of Location and Moving Path before Diagnosis.” The IPS content with the structures of FHIR resources is shown in Figure 7.

The Medication Summary section includes a description of the current and past medications that a patient takes. The Allergies or Intolerances section of a patient includes a description of the kind of reaction, the agents that caused it, as well as the criticality and the certainty of the allergy. The Problem List section includes clinical problems and the conditions of the patient that are currently being monitored. The Immunizations section includes a patient’s current immunization status and pertinent immunization history. The History of Procedures section includes a description of the patient procedures that are within the scope of the IPS. The Diagnostic Results section includes the relevant observations and in vitro biological specimens that are collected from the patient. In this section, the laboratory, imaging, and pathology reports may be included. The Vital Signs section includes the data collected when the patient received a medical service or was under surveillance in the hospital, such as the body temperature, blood pressure, heart rate, respiratory rate, height, weight, and BMI. The History of Illnesses section includes the patient’s disease history. This section can help physicians to make clinical decisions and get more information from the data. The Plan of Care section includes a description of the clinical care, such as a plan of the proposals, goals, monitoring, tracking, and ordering of requirements to improve the patient’s condition. The History of Location and Moving Path section includes where the patient has moved from and to during the incubation period of the infectious disease, as well as the location where the patient was infected (eg, a hospital, hotel, restaurant, bus, plane, or cruise ship). This section is important for controlling the spread of the disease, identifying potential patients, and completing prevention.

After the data of the FHIR IPS is uploaded, the system accepts the input by using the JavaScript Object Notation format. The FHIR IPS integrates each different resource into the same file as a “bundle” resource, and finally, it is uploaded into the HAPI server.

### Global COVID-19 Surveillance System for Case Studies

To achieve the purpose of global COVID-19 surveillance and to enhance health resilience, the exchange of global infectious disease information must be enacted. The COVID-19 surveillance system was built and designed based on the blockchain architecture. The IPS is used to exchange case study information among physicians. When physicians pass the system verification, they can upload the case IPS file and get the IPS data of other global cases from the system. The IPS file should be uploaded daily by the physician. The system includes daily IPS uploading and an enhancement plan, which covers real-time uploading through the interoperation of the clinic system with the module, based on the Open API architecture.

**Figure 8:**
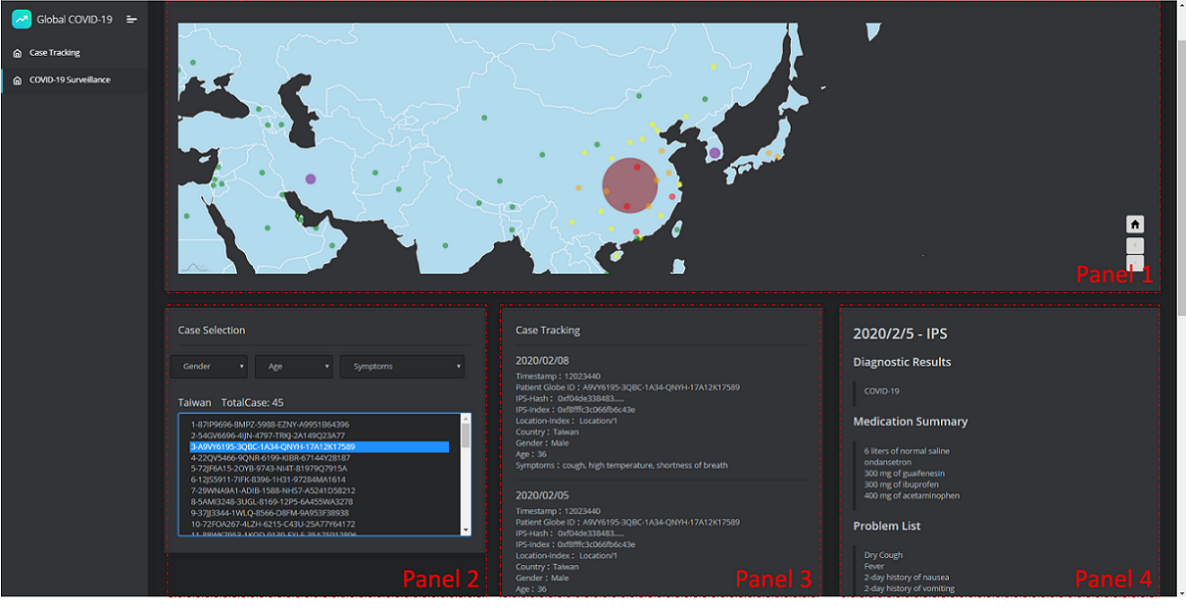
The COVID-19 surveillance system. IPS: International Patient Summary.

**Figure 9:**
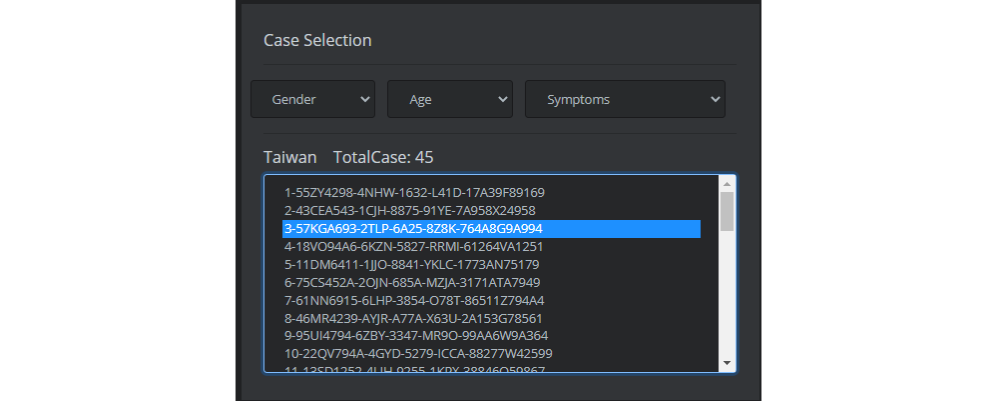
User interface of Case Selection (Panel 2).

All physician users have access to the case IPS files in the case study system to support clinical decision making. The system’s user interface (UI) is shown in Figure 8, and it is divided into four panels that achieve different functions. The case diagram is displayed in Panel 1, where users can obtain the number of cases and international case distribution information. Cases from different places can be selected in Panel 2, as well as in the system UI, as shown in Figure 9. The screening conditions are gender, age, and symptoms, which are used to screen-reference the cases that are similar to their own case. The case IPS information can be viewed in Panel 3, which includes all the uploaded IPS files, the basic information of the patient summary, and the IPS information on the blockchain. The detailed IPS content is viewed in Panel 4. The authenticated physician can use this system to share and exchange the patient IPS files to provide international references. Through the treatment of different cases, the drug treatments, and the exchange of the patient treatment results, the spread of the disease can be controlled, and treatment methods can be funded.

In our design, the user selects the country to track the case in Panel 1, and the country circle represents the number of cases. After selecting the country, Panel 2 will display the total number of case data that have been uploaded, as well as the GUID that each case represents in the system. Panel 2 gives the option to filter cases. After selecting a case, Panels 3 and 4 will display the IPS information of the selected case. The case selection (Panel 2) is shown in Figure 9. It is a Taiwanese example, and the patient GUID is represented as “5AIF63A5-9KWE-1653-AR1I-49682N29A22.”

### Blockchain Information of IPS Data

**Figure 10:**
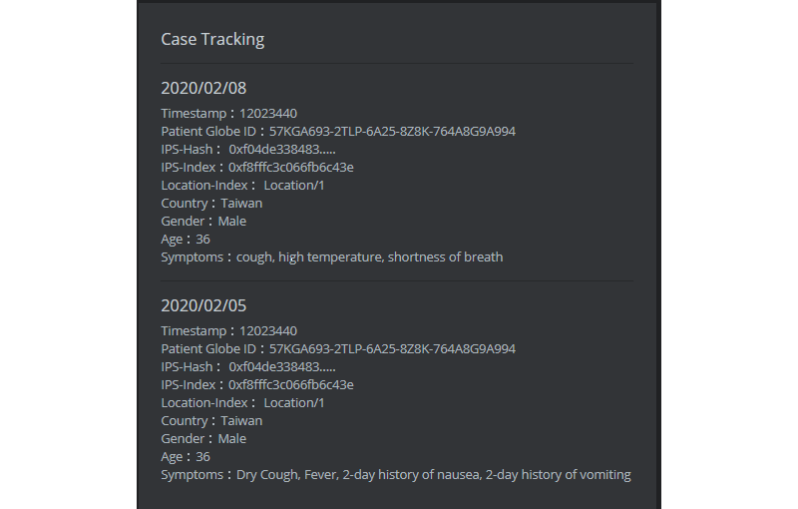
User interface of case tracking by block information.

In this study, all uploaded IPS information will be verified and stored in the uploading record by using the blockchain. Panel 3 is mainly the block information of the selected case. Figure 10 shows the block information of a patient whose GUID is “57KGA693-2TLP-6A25-8Z8K-764A8G9A994.” In the example, two blocks mean that the case has two uploaded IPS files, and the block information includes a time stamp, the GUID, and the IPS-hash and -index, as well as the moving location, country, gender, age, and symptoms.

### IPS File of COVID-19 Case

**Figure 11:**
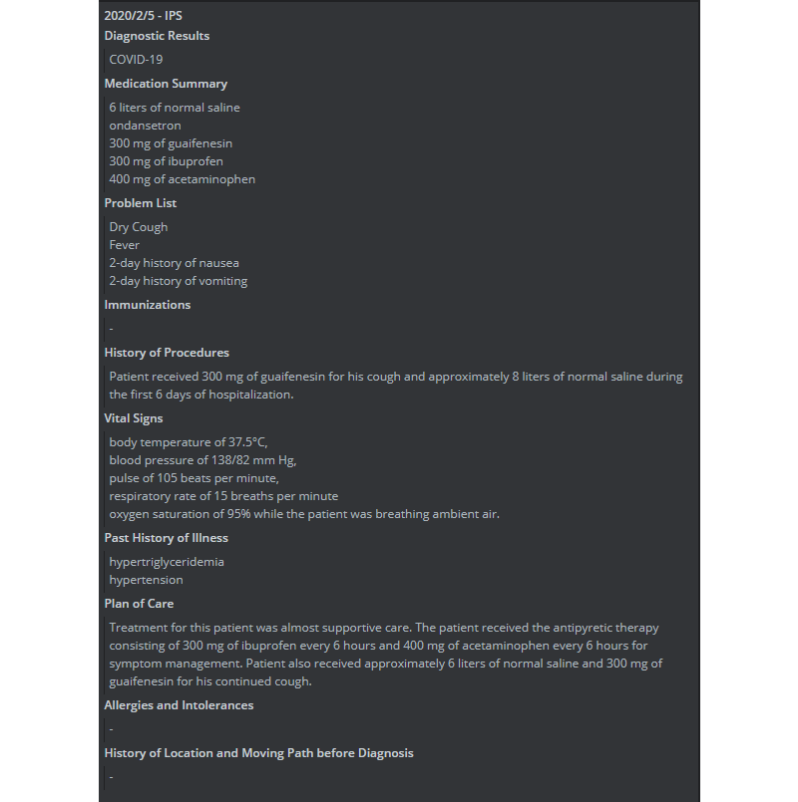
An example of a COVID-19 IPS file. IPS: International Patient Summary.

A COVID-19 case report is to be used as an example in this study. On February 5, 2020, a female patient 52 years of age presented with a fever and went to a hospital [28]. The patient had type 2 diabetes and had visited Wuhan on January 20. She developed a fever and myalgia 5 days after her return to Taiwan. She self-reported that she did not have dyspnea, a cough, chest pain, or diarrhea. The diagnosis of COVID-19 was made by a real-time reverse transcription polymerase chain reaction. The treatment for this patient was supportive care. The patient received the antipyretic therapy, which consisted of 300 mg of ibuprofen every 6 hours and 400 mg of acetaminophen every 6 hours for symptom management. The patient also received approximately 6 liters of normal saline and 300 mg of guaifenesin for her continued cough. An example of a COVID-19 IPS file is shown in Figure 11.

The following is additional information about the patient:

- Medication Summary
  6 liters of normal saline ondansetron
  300 mg of guaifenesin
  300 mg of ibuprofen
  400 mg of acetaminophen
- Problem List
  Dry cough
  Fever
  2-day history of nausea
  2-day history of vomiting
- Immunizations
- History of Procedures
  Patient received 300 mg of guaifenesin for her cough and approximately 8 liters of normal saline during the first 6 days of hospitalization.
- Vital Signs
  Body temperature of 37.5 °C
  Blood pressure of 138/82 mm Hg
  Pulse of 105 beats per minute
  Respiratory rate of 15 breaths per minute
  Oxygen saturation of 95% while the patient was breathing ambient air
- History of Illness
  Hypertriglyceridemia
  Hypertension

Based on other IPS files, international physicians can refer to the care plans of other patient, as well as their disease history, medication, and therapy, and give their own patients the appropriate therapy. Our system provides a new architecture for the exchange of IPS files.

### Case Tracking of COVID-19

**Figure 12:**
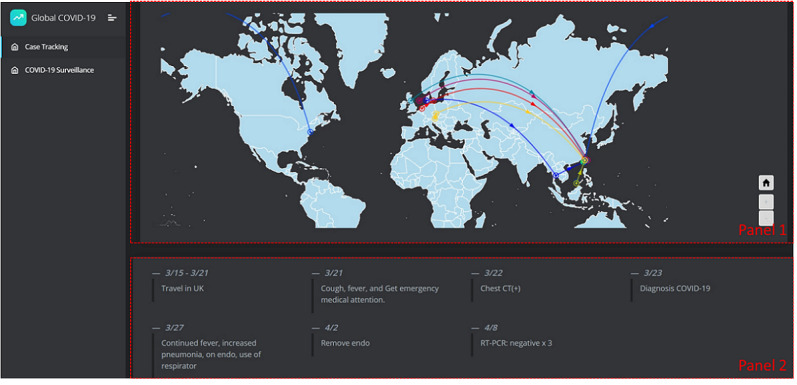
The COVID-19 Case Tracking system. Panel 1 shows the distributions of the moving paths of all the cases. Panel 2 shows the detailed moving path information and history record with the locations, time stamps, and possible activities. CT: computed tomography; RT-PCR: reverse transcription polymerase chain reaction.

From the establishment of the infectious disease case-tracking module, and by using the location information in the IPS file of the patient, we can track the moving paths of infectious disease cases. The location information of the patient is recorded in the block contents on the blockchain and is not protected as personal clinical data. Therefore, the location information can be retrieved and used by the system for the purpose of tracking the moving paths for different cases. The Case Tracking module has been established for CDC members to track cases and prevent the spread of a disease. Based on this module, CDC members can identify the moving paths of cases and design a case tracking plan for the epidemic investigation. The UI of the COVID-19 case tracking system is shown in Figure 12. The UI is divided into two panels. Nine cases that were diagnosed as COVID-19 in Taiwan were sampled as an example to show the case tracking function of the system. Their data were uploaded onto the blockchain, and the distributions of the moving paths of all cases is shown in Panel 1, where we can see all the worldwide cases as well as their moving paths in different colors on the map. The detailed case moving path information and history record is shown in Panel 2, with the locations, time stamps, and possible activities. In Panel 2 of Figure 12, we show the detailed information of one case. We can see that from March 15-21, 2020, the case had travelled in the United Kingdom. The case came back to Taiwan on March 21, showed some symptoms, and went to the emergency room. The case was confirmed as COVID-19 on March 23, and respirator use was started on March 27. From these nine samples, we can see that all of the cases were imported from outside of Taiwan.

## Discussion

After the outbreak of infectious diseases such as SARS, MERS, and COVID-19, it is well-known that international cooperation for disease treatment is critical, especially due to the current high frequency of travel between countries around the world. Diseases such as SARS and MERS not only affect people’s health but also seriously affect the world economy [29]. Although the deterioration of a disease condition depends on many variables, when facing unknown diseases, experience sharing and the exchange of advice are still key points. The control and treatment of any disease needs to be found as soon as possible. To control and treat the disease, a global case study sharing system must be established, not only for clinical data sharing but also for the development of treatment methods.

Through the system designed by this study, minimal and useful patient summary data can be shared. Physicians only need to focus on essential clinical data that can be followed up on, and they can try a specific treatment or medicine when facing unknown diseases such as COVID-19. Data from other countries or other patients can be taken as a reference for patient care and treatment. According to published studies, having a fever and a cough are the dominant symptoms of COVID-19, while gastrointestinal symptoms are uncommon [5,30,31]. One report presents the first confirmed case of COVID-19 in the United States, including the process of identification, diagnosis, clinical course, management, and the patient’s symptoms [3]. Overall, there is an important need for coordination between clinicians and public health authorities, as well as for the rapid transfer of clinical information relating to the care of patients with COVID-19.

One case study of the first-known imported case of COVID-19 infection in Taiwan describes how the doctor gave the patient supporting treatment for all her symptoms. However, there is still a lack of details on the clinical information about the patient [28]. Another study of numerous cases was conducted by Chan et al [32] at Hong Kong University. They found that the outbreak of COVID-19 in Wuhan, China was similar to the 2003 SARS outbreak in Guangzhou, China. Both outbreaks initially happened in the animal-to-people transmission model and not by person-to-person transmission in the community. The case study exchange from the model and the subsequent knowledge exchange, analysis, conclusion, planning, and evaluation will provide a basis for understanding the experiences of previous epidemics, like SARS and MERS, and help to streamline the disease prevention and control measures (eg, regulations for animal and wet markets, patient isolation and tracking, contact quarantine, and public health and hygiene education) to prevent any rapid spread. As their system was helpless against SARS, Hong Kong later developed the Communicable Disease Information System to provide real-time and intelligent syndromic and communicable disease surveillance; to enable rapid intervention and quicker outbreak and emergency responses via field investigations, outbreak control, responsive risk communication, ongoing analysis, alert generation, predictive capability, and early outbreak detection; and to offer a framework for strategic planning and program evaluation. We can rapidly gather information for COVID-19 through international channels, but the information is still not clear enough to use as a reference for treating patients. Lipsitch et al [33] showed that viral testing should not be used just for clinical care, and public health efforts should use it to target the trajectory and severity of the disease. Guan et al [34], from the State Key Laboratory of Respiratory Diseases, noted the limitations of COVID-19 research due to the collection of data from different structures of electronic databases and the urgent timeline for data extraction. Some cases, therefore, have incomplete clinical data of the patients’ exposure history and laboratory testing [34].

The main challenge of COVID-19 is that we do not have enough knowledge of the therapy, control methods, and full spread route of the virus, which can only be obtained from the patient. Based on the experience of rapid virus transmission and the burden on the health care system, a global information system is essential. When analyzing the development of COVID-19, it seems that an effective global communicable disease surveillance system has not yet been developed. The disease data are not timely or effectively linked. Physicians and scientists around the world are unable to obtain sufficient disease information in a thorough and timely manner to control the epidemic. Currently, the exchange of case data for clinical research on COVID-19 is incomplete and not quick enough, which limits the development of a treatment design. Even if many case reports were to be submitted, the goals of real-time tracking, data exchange, and referencing could not be achieved. Therefore, to reduce the restrictions on COVID-19 research, an EHRs–based information communication system is necessary, as it can quickly achieve such goals for the public.

This study created the IPS of infectious diseases that physicians can access when treating patients with COVID-19. We have also established a secure blockchain architecture for the protection of the IPS, and we have completed the application of tracking patients’ moving path. The IPS case studies can be exchanged through our system and verified through the blockchain architecture. Over the past few years, blockchain has been used in many different fields, not only with regard to medical records (EHRs and personal health records) but also to medical data exchange issues. Benil and Jasper [35] introduced blockchain architecture for managing EHRs. In its design, the EHR is stored in the cloud, and its integrity in the cloud will be checked through the blockchain. This is a similar architecture to our study and proves that the blockchain can protect and verify EHRs. Fan et al [36] proposed a blockchain-based consensus mechanism for medical information data security and privacy in the medical system. Sun et al [37] presented a distributed signature scheme for medical systems with a record-sharing protocol that is based on blockchain. Yang and Li [38] designed an architecture for securing the EHR system, which is based on distributed ledger technology, to improve the interoperability of health record exchanges between different organizations. Chen et al [39] introduced a searchable encryption scheme for EHRs by using blockchain. Blockchain architecture can ensure data security and verify that the information is correct, and it is therefore a suitable architecture for global IPS file exchange.

The results of this study can help health authorities respond quickly to the transmission and spread of any unknown disease, and it can provide a good system for information retrieval on disease transmission. Another benefit of this system is that it can help public health researchers form study trials and analyze data from different countries. A trial on medication treatment in patients with COVID-19 found that the lopinavir–ritonavir treatment added to the standard supportive care, but it was not significant for clinical improvement or mortality in patients with COVID-19 [40]. Other research on the use of chloroquine and hydroxychloroquine in COVID-19 shows that the use of these drugs is premature and potentially harmful [41].

However, the clinical observation details of patients were not described by the authors. It is hard to identify which supportive care works best for patients in different situations. Another effective means for fighting an unknown virus could be using a common forum to facilitate the mutual sharing of experiences, best practices, therapies for patients, and the possible useful medications and outcomes from clinical interventions being trialed in various countries in a secure, trustworthy manner. The system designed by this study can become an effective tool for facilitating global collaboration and cooperation, and for promoting collective evidence-based efforts to address the unprecedented situation created by COVID-19. However, this study has some limitations. At present, there is no optimal treatment, and complete information about this disease has not yet been found. Governments, medical institutions, and physicians from all over the world should cooperate in the study of this virus. Without international cooperation, global interests will have significant losses. This study has completed the design and development of a global infectious disease surveillance and case tracking system for COVID-19, and found that it has a stable foundation and is a balanced system. However, there is still a need to test the effectiveness of a large number of users uploading and exchanging data simultaneously. In the future, our team will have discussions with governments, international medical service providers, and medical institutions to activate this system and to promote international cooperation and development during the COVID-19 outbreak.

## Abbreviations

API: application programming interface
CDC: Centers for Disease Control and Prevention
EHR: electronic health record
FHIR: Fast Healthcare Interoperability Resources
GUID: globally unique identifier
HL7: Health Level 7
IPS: International Patient Summary
MERS: Middle East respiratory syndrome
PoA: Proof of Authority
SARS: severe acute respiratory syndrome
SEIPS: Systems Engineering Initiative for Patient Safety
UI: user interface
WHO: World Health Organization
